# Initial and ten-year treatment patterns among 11,000 breast cancer patients undergoing breast surgery—an analysis of German claims data

**DOI:** 10.1186/s12885-022-09240-w

**Published:** 2022-02-02

**Authors:** Miriam Heinig, Franziska Heinze, Sarina Schwarz, Ulrike Haug

**Affiliations:** 1 Department of Clinical Epidemiology, Leibniz Institute for Prevention Research and Epidemiology – BIPS, Achterstraße 30, 28359 Bremen, Germany; 2 Department of Health, Long-Term Care and Pensions, SOCIUM Research Center On Inequality and Social Policy, University of Bremen, 28359 Bremen, Germany; 3 Faculty of Human and Health Sciences, University of Bremen, Grazer Str. 2, 28359 Bremen, Germany

**Keywords:** Breast cancer, Cancer treatment, Claims data, Screening

## Abstract

**Background:**

We aimed to explore the potential of German claims data for describing initial and long-term treatment patterns of breast cancer patients undergoing surgery.

**Methods:**

Using the German Pharmacoepidemiological Research Database (GePaRD, ~ 20% of the German population) we included patients with invasive breast cancer diagnosed in 2008 undergoing breast surgery and followed them until 2017. We described initial and long-term treatment patterns and deaths. Analyses were stratified by stage (as far as available in claims data), age at diagnosis, and mode of detection (screen-detected vs. interval vs. unscreened cases).

**Results:**

The cohort comprised 10,802 patients. The proportion with neoadjuvant therapy was highest in patients < 50 years (19% vs. ≤ 8% at older ages). The proportion initiating adjuvant chemotherapy within four months after diagnosis decreased with age (< 50 years: 63%, 50–69: 46%, 70–79: 27%, 80 + : 4%). Among women < 69 years, ~ 30% had two breast surgeries in year one (70–79: 21%, 80 + : 14%). Treatment intensity was lower for screen-detected compared to interval or unscreened cases, both in year one (e.g., proportion with mastectomy ~ 50% lower) and within 2–10 years after surgery (proportions with radiotherapy or chemotherapy about one third lower each).

**Conclusions:**

This study illustrates the potential of routine data to describe breast cancer treatment and provided important insights into differences in initial and long-term treatment by mode of detection and age.

**Supplementary Information:**

The online version contains supplementary material available at 10.1186/s12885-022-09240-w.

## Background

Globally, breast cancer is the most common female cancer as well as the leading cause of cancer death among women [1]. In Germany, 68,950 women were newly diagnosed with breast cancer in 2016 (mean age 64 years), and 18,750 women died of breast cancer (mean age 75 years). The absolute five-year survival was 79% [2].

While the short- and long-term survival of breast cancer patients is well described by cancer registry data, there is scarcity of data on the burden of treatment, particularly regarding information beyond initial treatment [3–6]. Information on initial and long-term treatment is important given its impact on the quality of life among cancer patients [7, 8]. Stratification of this information by screening status would also be of interest given that screening is expected to reduce the intensity of treatment—apart from its expected impact on breast cancer mortality. However, few studies have compared treatment between screen-detected vs. not screen-detected breast cancer cases [9–12].

In Germany, long-term information on cancer treatment is not available from cancer registries yet as clinical cancer registration was established only recently [13]. One cancer registry has already been collecting treatment information on about 8,500 breast cancer patients followed for an average of ten years but follow-up data was missing in about 18–44% of patients (depending on the type of treatment) [14]. To fill this gap, German claims data may be a valuable data source but they have not been used for this purpose so far. Claims data also contain information that can be used to classify breast cancer patients into screen-detected vs. not screen-detected cases. In Germany, women aged 50–69 years are invited biennially to participate in the organized mammography screening program.

Our aim was therefore to explore the potential of German claims data for describing the initial and long-term treatment of breast cancer patients who underwent breast surgery, stratified by baseline characteristics of the disease as far as available in claims data and the mode of detection (screen-detected vs. not screen-detected).

## Methods

### Data source

We used the German Pharmacoepidemiological Research Database (GePaRD) which is based on claims data from four statutory health insurance providers in Germany. GePaRD currently includes information on approximately 25 million persons who have been insured with one of the participating providers since 2004 or later. In addition to demographic data, GePaRD contains information on drug dispensations as well as outpatient (i.e., from general practitioners and specialists) and inpatient services and diagnoses. Per data year, there is information on approximately 20% of the general population and all geographical regions of Germany are represented. About 90% of the general population in Germany are covered by statutory health insurance. Core characteristics of the German health insurance system are uniform access to all levels of care and free choice of providers. As detailed in Additional file 1, various measures are taken to ensure a high quality and to assess validity of the data in GePaRD.

### Study design

Given our aim to provide information on initial treatment (starting before or within one year after surgery as further explained below) and long-term treatment (years 2–10 after breast surgery), we conducted a retrospective cohort study among female breast cancer patients diagnosed in 2008. We included women with a first diagnosis code for breast cancer (ICD-10-GM “C50”, inpatient or outpatient code) in 2008 and a second breast cancer code within four months to confirm the diagnosis. We followed the cohort until death or the end of observation on December 31, 2017. Another inclusion criterion was continuous health insurance coverage for four years before the breast cancer diagnosis code in 2008. We excluded patients with a code for breast cancer during this pre-observation period to focus the analysis on incident breast cancer cases. Furthermore, we excluded patients without breast surgery and patients who had no inpatient diagnosis code for “C50” but an inpatient diagnosis code for in situ carcinoma of the breast (ICD-10-GM “D05”) in the quarter of surgery. Finally, we excluded patients who left the cohort due to a change of insurance provider, i.e. with incomplete follow-up. While we included breast cancer patients irrespective of age into the cohort, the analysis by mode of detection was restricted to those in the age range eligible for screening.

### Classification of stage at diagnosis and mode of detection

Given that exact information on tumor stage is not available in claims data, we used a previously developed algorithm to roughly classify stage at diagnosis as far as possible in claims data [15]. This algorithm considers in- and outpatient diagnoses for secondary neoplasms (ICD-10-GM “C77”, “C78”, “C79”) coded during the 120 days following the incident breast cancer diagnosis and classifies patients into three categories: no affected lymph nodes or distant metastases at diagnosis (group **A**), only affected lymph nodes at diagnosis (group **B**), and distant metastases at diagnosis (group **C**).

Regarding the mode of detection, we distinguished between “screen-detected”, “interval cancer”, “unscreened, but eligible” and “unscreened and ineligible”. These categories take into account that in Germany women are eligible to participate in mammography screening from age 50 to 69 every two years. To define “screen-detected”, we considered codes for screening mammography and multidisciplinary case conference in relevant time periods as explained in Additional file 1. Multidisciplinary case conferences are only supposed to be held for patients with a biopsy taken after suspicious findings at screening mammography, so they also indicate screen detection. The diagnosis was classified as “interval cancer” if the woman had a screening mammography in the regular screening interval (two years) before diagnosis, but the criteria for screen-detection were not fulfilled. If no mammography screening was coded in the regular screening interval before diagnosis and the woman was eligible for screening (i.e., 50–69 years at diagnosis), the patient was classified as “unscreened but eligible”. The remaining patients were classified as “unscreened and ineligible”. A figure describing this classification is available in Additional file 1 (Figure A1).

### Description of breast cancer treatment and deaths

We assessed breast cancer treatment using claims for in- and outpatient medication (systemic therapy, i.e. cytostatic drugs, hormone therapy, monoclonal antibodies) and claims for in- and outpatient procedures (breast surgery, radiotherapy). To describe initial therapy we used the following time frames and categories: proportion of patients with cancer medication initiated after diagnosis and before surgery (neoadjuvant systemic therapy), proportion of patients with cancer medication initiated within four months after breast surgery (adjuvant systemic therapy), and proportion of patients with radiotherapy initiated within ten months after breast surgery. We described initial therapy stratified by stage at diagnosis (groups **A**–**C**) and also determined the proportion of deaths during initial treatment and afterwards. Furthermore, we described initial therapy stratified by age at diagnosis (< 50 years, 50–69 years, 70–79 years, 80 + years) and stratified by mode of detection among women eligible for mammography screening, i.e. 50–69-year-old women (screen-detected cancer, interval cancer, cancer in unscreened women). Regarding long-term treatment, we described treatment as well as deaths in the years 2–10 after first breast surgery, determining for each year the proportion of patients with at least one code for radiotherapy, cytostatic drugs, or further breast surgery (i.e., breast conserving surgery/mastectomy; codes for breast reconstruction were not considered) in the respective year and the proportion of women who died in the respective year. In addition, we determined the proportion of patients with any code for radiotherapy, cytostatic drugs, or further breast surgery or death considering the years 2–10 together.

We conducted descriptive analyses, calculating proportions of women who received the respective treatment or died, and means and standard deviations to describe age. All analyses were performed using SAS® software (SAS Institute, version 9.4, NC, USA).

## Results

The initial cohort included 12,704 breast cancer patients diagnosed in 2008 who received breast surgery within one year after diagnosis. Of those, 1,902 were excluded because they only had an inpatient code for in situ carcinoma of the breast in the quarter of surgery (*n* = 674) or had an incomplete follow-up due to a switch in insurance (*n* = 1228), leaving a final cohort of 10,802 women. The median duration of follow-up was ten years (interquartile range 9–10 years).

### Initial treatment patterns

**Fig. 1:**
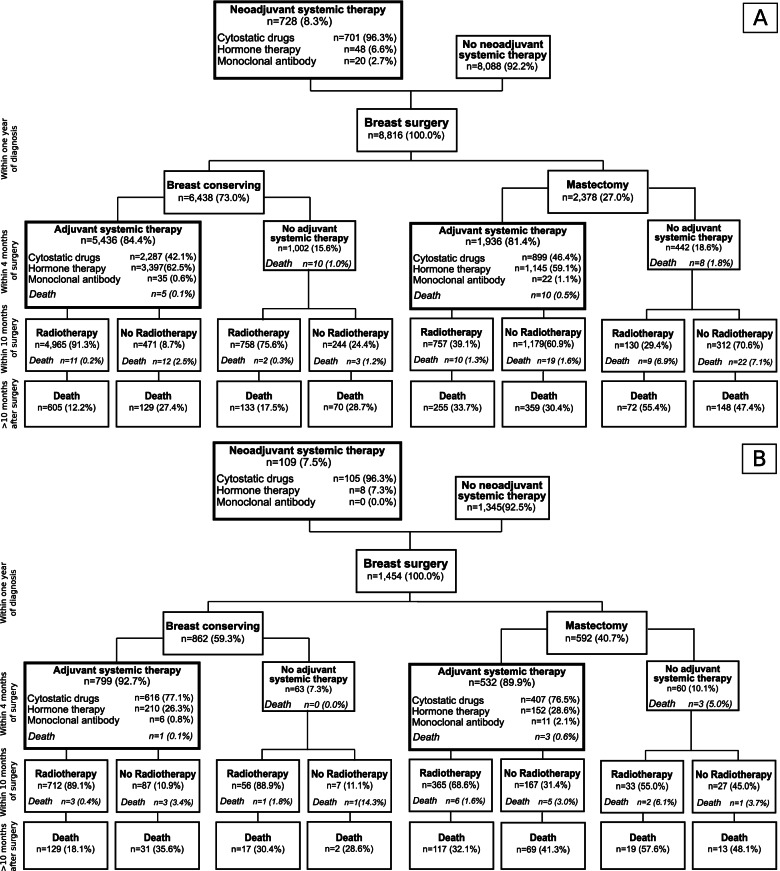
Description of initial treatment phase in included breast cancer patients without affected lymph nodes or distant metastases (**A**) or with affected lymph nodes only (**B**) at diagnosis. The time frames considered for treatment and death within treatment groups were: breast conserving surgery or mastectomy within one year after diagnosis, adjuvant systemic therapy initiated within four months after diagnosis, and radiotherapy initiated within ten months after diagnosis. Deaths after the initial treatment phase were assessed later than ten months after diagnosis

**Fig. 2:**
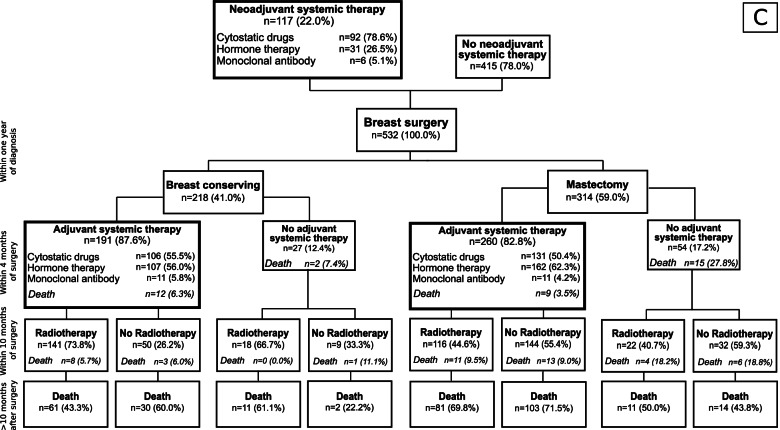
Description of initial treatment phase in included breast cancer patients with distant metastases (**C**) at diagnosis. The time frames considered for treatment and death within treatment groups were: breast conserving surgery or mastectomy within one year after diagnosis, adjuvant systemic therapy initiated within four months after diagnosis, and radiotherapy initiated within ten months after diagnosis. Deaths after the initial treatment phase were assessed later than ten months after diagnosis

Figure 1 and Fig. 2 show the treatment patterns before and within ten months after surgery, stratified by the presence or absence of codes regarding affected lymph nodes or distant metastases at the time of diagnosis. Overall, group **A**, **B**, and **C** comprised 8,816, 1,454, and 532 patients, respectively. The proportion of patients receiving systemic cancer therapy before surgery was 8% in group **A** and **B** and 22% in group **C**. In group **A**, the proportion of patients with a mastectomy in the first year after breast cancer diagnosis was 27% (group **B**: 41%; group **C**: 59%). The proportion of patients starting adjuvant systemic therapy within four months after surgery was about 80% in group **A**, about 90% in group **B**, and between 83 and 88% in group **C**. In all three groups, but particularly in group A, the proportion of patients starting radiotherapy within ten months after surgery was higher in patients with breast conserving surgery (BCS) than in patients with a mastectomy. For example, in patients of group **A**, 91% of patients who received BCS and adjuvant systemic therapy started radiotherapy within ten months after surgery, vs. 39% of patients with mastectomy and adjuvant systemic therapy. In patients of group **B**, these proportions were 89% vs. 69%.

### Initial treatment by age at diagnosis

Table 1 describes patient characteristics and initial cancer treatment by age at diagnosis. The proportion of patients with BCS was highest in age group 50–69 (76%) and lowest in patients aged 80 or older (38%). Both the proportion of patients with a second breast surgery within one year after first surgery and with neoadjuvant treatment were highest in patients below age 50 (31% and 19%, respectively). The proportion of patients initiating treatment with cytostatic drugs within four months after surgery was 63% below age 50, 46% in age group 50–69, 27% in age group 70–79, and 4% in patients aged 80 or older. The initiation of hormone therapy within four months after surgery varied between 30% (< age 50), 46% (ages 50–69), 59% (ages 70–79), and 65% (age 80 +). Radiotherapy was initiated in 74%–80% of patients within ten months after breast surgery, except for age group 80 or older (34%).

**Table 1:** Characterization of included breast cancer patients and description of initial treatment phase by age at diagnosis

	**All**		**Age at diagnosis**							
			** < 50 years**		**50–69 years**		**70–79 years**		**80 + years**	
	10,802	(100%)	1750	(16.2%)	5950	(55.1%)	2119	(19.6%)	983	(9.1%)
Mean age at diagnosis (SD)	62.8	(12.2)	43.9	(4.5)	61.0	(5.9)	73.6	(2.8)	84.1	(3.6)
**Stage at diagnosis**										
No affected lymph nodes/distant metastases	8816	(81.6%)	1419	(81.1%)	4910	(82.5%)	1702	(80.3%)	785	(79.9%)
Affected lymph nodes only	1454	(13.5%)	266	(15.2%)	781	(13.1%)	284	(13.4%)	123	(12.5%)
Distant metastases	532	(4.9%)	65	(3.7%)	259	(4.4%)	133	(6.3%)	75	(7.6%)
**Breast surgery**^**a**^										
Breast conserving surgery only	7518	(69.6%)	1234	(70.5%)	4511	(75.8%)	1401	(66.1%)	372	(37.8%)
Mastectomy	3284	(30.4%)	516	(29.5%)	1439	(24.2%)	718	(33.9%)	611	(62.2%)
Both types of surgery	1078	(10.0%)	222	(12.7%)	605	(10.2%)	179	(8.4%)	72	(7.3%)
Two or more surgeries	2716	(25.1%)	543	(31.0%)	1588	(26.7%)	452	(21.3%)	133	(13.5%)
**Neoadjuvant systemic therapy**										
Yes	954	(8.8%)	330	(18.9%)	497	(8.4%)	101	(4.8%)	26	(2.6%)
**Adjuvant systemic therapy**^**b**^										
Cytostatic drugs	4446	(41.2%)	1101	(62.9%)	2723	(45.8%)	581	(27.4%)	41	(4.2%)
Monoclonal antibody	96	(0.9%)	18	(1.0%)	55	(0.9%)	20	(0.9%)	3	(0.3%)
Hormone therapy	5173	(47.9%)	524	(29.9%)	2758	(46.4%)	1257	(59.3%)	634	(64.5%)
**Radiotherapy**^**c**^										
Within ten months after breast surgery	8073	(74.7%)	1398	(79.9%)	4773	(80.2%)	1565	(73.9%)	337	(34.3%)
Before breast surgery	58	(0.5%)	9	(0.5%)	38	(0.6%)	9	(0.4%)	2	(0.2%)

*SD* standard deviation, *N/A* not applicable

^a^Within one year after diagnosis. Mastectomy includes those with both types of surgery. “Two or more surgeries” refers to additional breast conserving surgery/mastectomy in the first year after the first surgery

^b^This refers to adjuvant systemic therapy initiated within four months after breast surgery

^c^This refers to radiotherapy initiated within ten months after breast surgery

### Initial treatment by mode of detection

Table 2 describes patient characteristics and initial therapy stratified by mode of detection in screening-eligible patients. Overall, 34% were classified as screen-detected and 8% were classified as interval-detected. The proportion in stage group A (no codes for affected lymph nodes or metastases) was highest among screen-detected patients (88%) and lowest among unscreened patients (80%). BCS was most common among screen-detected patients (86% vs. 73% and 71% in the interval and unscreened group, respectively). In all three groups, 24–31% had a second breast surgery within one year after first surgery. The proportion of patients with neoadjuvant systemic therapy was lowest in screen-detected patients (2% vs. 9–12%). The proportion of patients initiating treatment with cytostatic drugs within four months after surgery was also lowest in this group (38% vs. 50%). Hormone therapy was most common among screen-detected patients (53% vs. 42–43%).

**Table 2:** Characterization of included breast cancer patients who were eligible for screening and description of initial treatment phase by mode of detection

	**All**		**Mode of detection**					
			**Screening participants**				**Unscreened (eligible)**	
			**Screen-detected**^**a**^		**Interval-detected**			
	6065	(100%)	2049	(33.8%)	476	(7.8%)	3540	(58.4%)
**Age at diagnosis**								
Mean age at diagnosis (SD)	61.2	(6.0)	62.0	(5.8)	61.9	(6.0)	60.6	(6.0)
< 50 years at diagnosis	N/A	(0%)	N/A	(0%)	N/A	(0%)	N/A	(0%)
50–69 years at diagnosis	5950	(98.1%)	1970	(96.1%)	440	(92.4%)	3540	(100%)
70–79 years at diagnosis	115	(1.9%)	79	(3.9%)	36	(7.6%)	N/A	(0%)
80 + years at diagnosis	N/A	(0%)	N/A	(0%)	N/A	(0%)	N/A	(0%)
**Stage at diagnosis**								
No affected lymph nodes/distant metastases	5015	(82.7%)	1807	(88.2%)	392	(82.4%)	2816	(79.5%)
Affected lymph nodes only	788	(13.0%)	206	(10.1%)	72	(15.1%)	510	(14.4%)
Distant metastases	262	(4.3%)	36	(1.8%)	12	(2.5%)	214	(6.0%)
**Breast surgery**^**b**^								
Breast conserving surgery only	4608	(76.0%)	1756	(85.7%)	348	(73.1%)	2504	(70.7%)
Mastectomy	1457	(24.0%)	293	(14.3%)	128	(26.9%)	1036	(29.3%)
Both types of surgery	610	(10.1%)	151	(7.4%)	61	(12.8%)	398	(11.2%)
Two or more surgeries	1606	(26.5%)	494	(24.1%)	145	(30.5%)	967	(27.3%)
**Neoadjuvant systemic therapy**								
Yes	501	(8.3%)	43	(2.1%)	43	(9.0%)	415	(11.7%)
**Adjuvant systemic therapy**^**c**^								
Cytostatic drugs	2758	(45.5%)	771	(37.6%)	236	(49.6%)	1751	(49.5%)
Monoclonal antibody	57	(0.9%)	6	(0.3%)	4	(0.8%)	47	(1.3%)
Hormone therapy	2823	(46.5%)	1086	(53.0%)	200	(42.0%)	1537	(43.4%)
**Radiotherapy**^**d**^								
Within ten months after breast surgery	4866	(80.2%)	1708	(83.4%)	381	(80.0%)	2777	(78.4%)
Before breast surgery	38	(0.6%)	3	(0.1%)	2	(0.4%)	33	(0.9%)

*SD* standard deviation, *N/A* not applicable

^a^Breast cancer was classified as “screen-detected” if a screening mammography and multidisciplinary case conference were coded in relevant time periods before and surrounding the diagnosis. It was classified as “interval-detected” if the woman had a screening mammography in the regular interval (two years) before diagnosis, but the criteria for “screen-detected” were not fulfilled. Patients without a screening mammography in the regular interval and aged 50–69 years at diagnosis were classified as unscreened, but eligible. The remaining patients were classified as “unscreened and ineligible” (not included in this table).Some patients may be diagnosed, e.g., at age 70 and screened at age 69

^b^Within one year after diagnosis. Mastectomy includes those with both types of surgery. “Two or more surgeries” refers to additional breast conserving surgery/mastectomy in the first year after the first surgery

^c^This refers to adjuvant systemic therapy initiated within four months after breast surgery

^d^This refers to radiotherapy initiated within ten months after breast surgery

The initial treatment stratified by stage at diagnosis is available in Additional file 1 (Table A1).

### Long-term treatment patterns and deaths

**Fig. 3:**
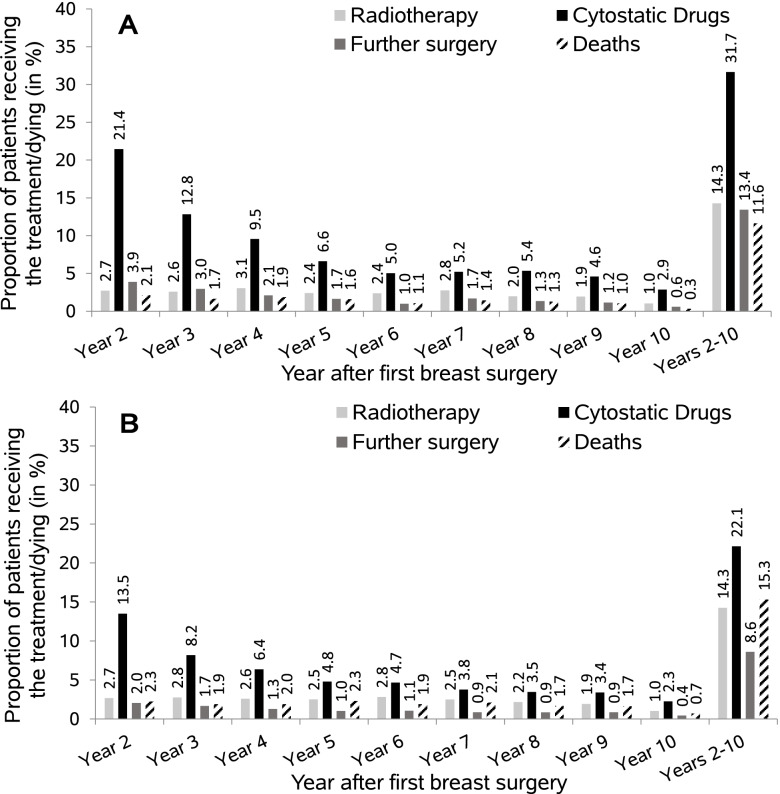
Long-term treatment patterns (radiotherapy, cytostatic drugs, further surgery [breast conserving surgery/mastectomy]) and deaths in the years 2–10 among included breast cancer patients stratified by age group (< 50 years (**A**) and 50–69 years (**B**) at diagnosis). Year ten is not a full year because observation ended on December 31, 2017

Figure 3 shows the long-term treatment patterns and deaths in the years 2–10 after first breast surgery for patients below age 50 and patients aged 50–69. In patients aged 50–69, the proportion receiving chemotherapy and the proportion with another surgery declined from year two (chemotherapy: 13.5%, surgery: 2.0%) to year five (chemotherapy: 4.8%, surgery: 1.0%) and showed little variation afterwards. In women below age 50, a similar pattern was observed but the proportions were higher (year 2: 21.4% with chemotherapy, 3.9% with surgery; year 5: 6.6% with chemotherapy, 1.7% with surgery). The proportion of women who died was rather similar in both age groups by year four, while from year five onwards, it was 0.4–0.9 percentage points higher in women aged 50–69. Overall, the proportion of patients ever receiving chemotherapy in the years 2–10 was higher by about ten percentage points in patients below age 50 (further surgery: 5 percentage points higher). Ever treatment with a monoclonal antibody in the years 2–10 was more common in patients below age 50 (12% vs. 8%) while ever treatment with hormone therapy was less common in this age group (72% vs. 80%) (data not shown). For women aged 70–79 and 80 years and older, the long-term treatment patterns and proportion of deaths are shown in Additional file 1 (Figure A2).

**Fig. 4:**
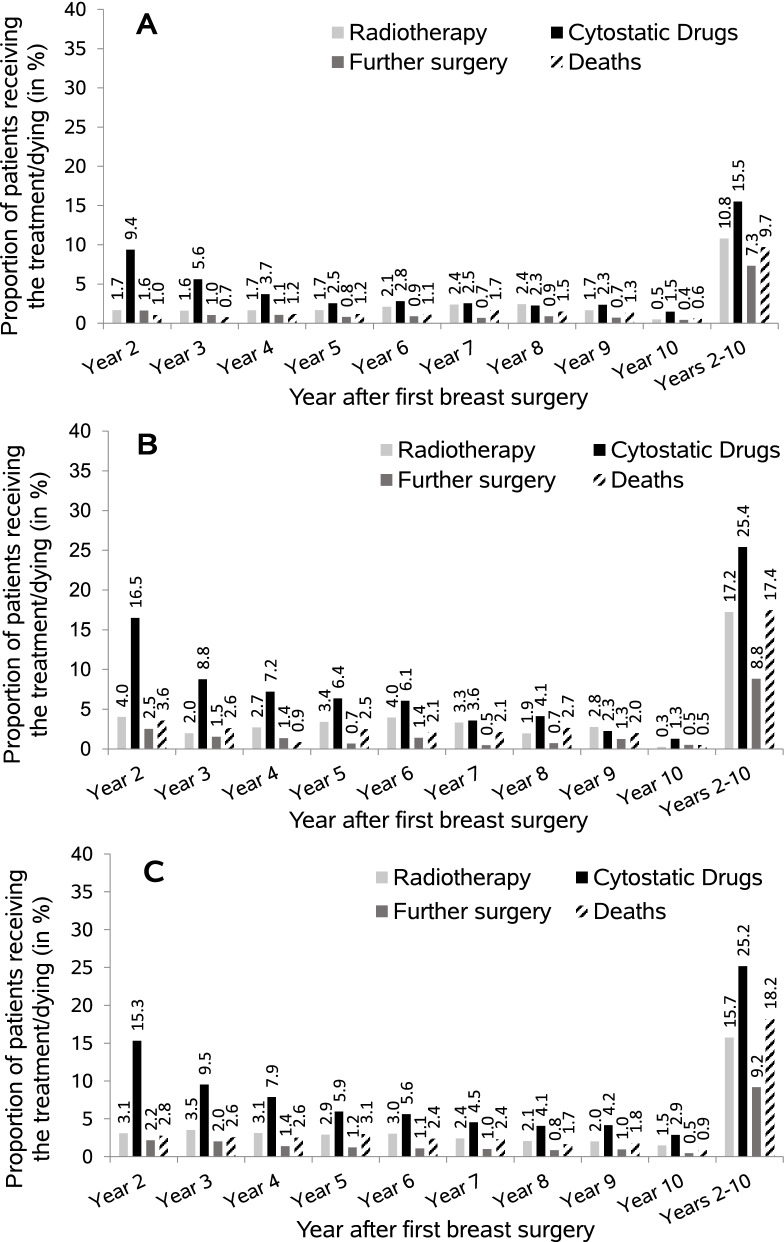
Long-term treatment patterns (radiotherapy, cytostatic drugs, further surgery [breast conserving surgery/mastectomy]) and deaths in the years 2–10 after first breast surgery in screening-eligible patients whose breast cancer was screen-detected (**A**), detected in the screening interval (**B**), or who were eligible but did not participate in screening (**C**). Year ten is not a full year because observation ended on December 31, 2017

Figure 4 shows the long-term treatment patterns and deaths in the years 2–10 after first breast surgery by mode of detection among screening-eligible women (50–69 years). From year two to year nine the proportions of women receiving treatment or who died were generally higher for interval cancers as compared to screen-detected cancers. From year two to year eight the proportions of women receiving cytostatic drugs were 29–60% higher among interval cancers. For radiotherapy, the differences were highest in years two, five and six. The proportions of patients receiving further surgery also tended to be higher among interval cancers in most years. Among unscreened women, the long-term treatment patterns were rather similar to women with interval cancers. Overall, the proportion of patients ever receiving cytostatic drugs in the years 2–10 was higher by about ten percentage points among interval-detected patients as compared to screen-detected cancers (25.4% vs. 15.5%). The proportion of patients receiving radiotherapy in the years 2–10 was 11–12% among screen-detected patients irrespective of the type of surgery in the first year. Among interval-detected patients this proportion was higher than among screen-detected patients and further differed according to the type of surgery in the first year (mastectomy: 22%; BCS: vs. 16%). Ever treatment with a monoclonal antibody in the years 2–10 was less common for screen-detected cancers as compared to interval cancers (5% vs. 8–9%) while ever treatment with hormone therapy was more common in this group (84% vs. 75–80%) (data not shown).

The long-term treatment patterns and deaths for the whole cohort and stratified by stage at diagnosis are available in Additional file 1 (Figures A3 and A4).

## Discussion

To the best of our knowledge, this is the first study providing detailed and complete information on initial and long-term treatment patterns and deaths for a large group of breast cancer patients in Germany. Including 10,802 patients diagnosed in 2008 who underwent breast surgery, we observed a markedly lower intensity of treatment for screen-detected breast cancer cases as compared to cases detected in the screening interval or among unscreened women eligible for screening, both in the first year (e.g., ~ 50% lower proportion of patients with mastectomy) and in the years 2–10 after diagnosis (proportions of patients with radiotherapy or chemotherapy about one third lower each). We also observed distinct differences in treatment by age at diagnosis. The proportion of patients initiating chemotherapy within four months after surgery, for example, declined from 63% in patients below age 50 to 27% in patients aged 70–79 and was only 4% in patients aged 80 or older. Among women aged 69 or younger, ~ 30% had a second breast surgery in the first year after first surgery; among women aged 70–79 years and 80 years or older, this proportion was still 21% and 14%, respectively.

The lower intensity of treatment among screen-detected breast cancers was expected given that screening leads to a more favorable stage distribution. This lower treatment intensity may not fully be interpreted as a benefit of screening as there are likely a certain proportion of overdiagnosed cancers in this group even though the extent of overdiagnosis in breast cancer screening is a matter of ongoing debate [16]. There is hardly any other study that quantified and compared the initial and long-term intensity of treatment in screened vs. interval and unscreened breast cancers. Existing studies only provided information on initial treatment and even this information was often restricted to certain types of treatment, particularly surgery and chemotherapy [9, 10, 17–20]. In addition, they commonly included ductal carcinoma in situ (DCIS) [17, 19, 21, 22], which amounted to 15–30% of screen-detected cases in some studies [17, 22]. A study from Germany by Braun et al. reported that 77% of 526 screen-detected vs. 67% of 147 interval-detected cases with invasive breast cancer underwent BCS as primary surgical treatment [12]. In our study a similar difference between both groups was observed but the proportions of patients undergoing only BCS were higher compared to the study by Braun et al., particularly for screen-detected cases (86%). This is likely due to the fact that Braun et al. included cases since 2006, i.e., a high proportion was detected in the first round of screening where stage distribution is typically less favorable as compared to subsequent rounds.

We observed a higher proportion of women initiating hormone therapy within four months after surgery among screen-detected cancer cases (53%) as compared to interval and unscreened cases (42–43%). Long-term receipt of hormone therapy was also higher in this group (84% vs. 75–80%, respectively). This is in line with other studies reporting a higher proportion of hormone receptor-positive breast cancer in this group [23, 24]. For example, in the study by Braun et al., this proportion was 78%, 70%, and 68% among screen-detected, interval, and unscreened cases, respectively [12]. In a systematic review, we have shown that women using menopausal hormone therapy are more adherent to mammography screening as compared to non-users [25]. This association in combination with the fact that menopausal hormone therapy increases breast cancer risk, particularly risk of hormone receptor-positive breast cancer [26, 27], may explain this imbalance between groups.

In the youngest age group (< 50 years at diagnosis), we observed the highest proportions of women receiving neoadjuvant systemic therapy and adjuvant chemotherapy. This may reflect a higher proportion of larger, more advanced cancers at diagnosis (size was not captured in our data). On the other hand, a more aggressive treatment even for stage 1 in breast cancer patients below age 40 compared to older patients has been reported by Fredholm et al. [28]. Further, receipt of both types of surgery (BCS and mastectomy) was also most common in the youngest age group (13% vs. 7–10% in the older age groups). This might indicate that the initial treatment decision in this age group is driven by the aim to conserve the breast, more so than in other age groups, but treatment has then to be intensified in a second step. From a data perspective, this highlights the importance of collecting information beyond initial treatment in order to avoid underestimating treatment intensity. Conversely, we observed the highest proportion of mastectomies and the lowest proportions of treatment (further surgery, neoadjuvant and adjuvant systemic treatment, radiotherapy) among patients aged 70 years or older, particularly in those aged 80 + . This is likely due to higher comorbidity, frailty, or different patient preferences in the older compared to the younger age groups. Nonetheless, there have also been concerns that breast cancer patients with comorbidity are not optimally treated [29]. Our finding is consistent with a German study from 2014 reporting a lower proportion of patients receiving BCS, chemotherapy, and targeted therapy for patients aged 70 years or older as compared to those aged 15–69 years [30]. Regarding the type of surgery, a study from the UK among breast cancer patients 70 years or older undergoing surgery also reported that the proportion of patients with a mastectomy (as opposed to BCS) increased with age [31].

Our analysis regarding the long-term treatment patterns in the years 2–10 following the first breast surgery provides information on the burden of treatment beyond initial treatment. Furthermore, it may provide indicators for recurrence, but this needs to be interpreted with caution because, for example, in year two the initial treatment may not be finished yet or in very advanced patients, initial treatment may continue until death. The proportions of patients with further treatment (radiotherapy, cytostatic drugs, surgery) were higher among interval-detected and unscreened patients compared to screen-detected patients. This difference was particularly pronounced for cytostatic drugs. Stratification by age showed that the proportions of patients with further treatment in the years following the first breast surgery were highest among the youngest age group (< 50 years at diagnosis), which might indicate elevated rates of recurrence. To which extent this is due to a less favorable stage at diagnosis or a more progressive nature of the tumors needs to be clarified but this requires additional clinical and molecular data.

A specific strength of our study lies in the data source which allowed complete and long-term follow-up of cancer treatment in a large sample of breast cancer patients representing the real-world setting. The data source avoids non-responder and recall bias and allowed stratification by mode of detection (screen-detected vs. interval vs. unscreened cases). Furthermore, it has also been shown that drug prescriptions in GePaRD are representative of all persons with statutory health insurance in Germany [32]. To make sure that the study population can be followed up for ten years and is as homogeneous as possible with respect to treatment guidelines at the time of diagnosis, we focused on one year of diagnosis (2008) regarding inclusion. We plan on repeating this analysis for further years of diagnosis as soon as sufficient follow-up data is available in GePaRD.

Some limitations should also be kept in mind. First, claims data are limited with respect to information on stage at diagnosis. We still tried to roughly distinguish between more and less advanced stages and found plausible patterns, but we are aware that this does not correspond to the usual stage classification of breast cancer. Regarding the breast cancer diagnosis itself, we applied strict criteria for case definition, so we consider it very unlikely that women who did not actually have breast cancer were included. Second, there is a lack of information on molecular tumor characteristics, but a feasibility study exploring the potential of claims data to roughly assess receptor status is ongoing, which might be used for future analysis. Future studies may also assess whether it is possible to determine detailed treatment regimen (e.g., chemotherapy regimen) in claims data. Third, as we only included patients who underwent surgery within one year of diagnosis no conclusions from our study can be drawn regarding breast cancer patients not treated surgically. Lastly, the case definition in our study required four years of continuous health insurance before diagnosis in order to distinguish incident from prevalent breast cancers. However, given the features of the German health system (e.g., insurance coverage is independent of occupational status) and also in view of the fact that switching health insurance provider is uncommon in Germany, we do not think that this inclusion criterion resulted in a selected group of patients.

## Conclusions

In conclusion, this study illustrates the potential of claims data in providing long-term data on breast cancer treatment in the real-life setting and provided important insights, amongst others, into differences in initial- and long-term treatment by mode of detection and age.

## Supplementary Information

**Additional file 1.**

## Data Availability

As we are not the owners of the data we are not legally entitled to grant access to the data of the German Pharmacoepidemiological Research Database. In accordance with German data protection regulations, access to the data is granted only to employees of the Leibniz Institute for Prevention Research and Epidemiology – BIPS on the BIPS premises and in the context of approved research projects. Third parties may only access the data in cooperation with BIPS and after signing an agreement for guest researchers at BIPS.

## Abbreviations

BCS: Breast conserving surgery
GePaRD: German Pharmacoepidemiological Research Database
ICD-10-GM: International Classification of Diseases – 10. Revision – German Modification
DCIS: Ductal carcinoma in situ
