# Supplementary material for: Initial and ten-year treatment patterns among 11,000 breast cancer patients undergoing breast surgery—an analysis of German claims data
Source: BMC Cancer. 2022 Feb 2;22:130. doi: 10.1186/s12885-022-09240-w (PMC8812022; doi:10.1186/s12885-022-09240-w)
Supplement: Supplementary file 1 — Additional file 1. [file 12885_2022_9240_MOESM1_ESM.pdf]

Additional information to: Initial and ten-year treatment patterns among 11,000 breast cancer patients undergoing breast surgery—an analysis of German claims data

Miriam Heinig, Franziska Heinze, Sarina Schwarz, Ulrike Haug

Measures to ensure a high data quality in the German Pharmacoepidemiological Research Database (GePaRD)

Figure A1: Visualization of the definition of mode of detection

Table A1: Patient characteristics and initiation of first cancer treatment by stage at diagnosis

Figure A2: Long-term treatment patterns and deaths in patients aged 70-79 years and 80 years and older

Figure A3: Long-term treatment patterns and deaths in all patients

Figure A4 A-C: Long-term treatment patterns and deaths by stage at diagnosis

Measures to ensure a high data quality in the German Pharmacoepidemiological Research Database (GePaRD)

In GePaRD, we have established standard operating procedures and take several measures to ensure a high data quality. When we receive the data there are extensive plausibility checks including comparison with prior data years to detect inconsistencies or incompleteness in the data. Any issues are then resolved in dialogue with the respective health insurance provider. When we analyze the data, we take into account that there are types of data that have a high validity due to strict coding guidelines (hospital data) or automated processes for data entry (pharmacy data) but also types of data with a lower degree of validity such as outpatient diagnosis. When we use outpatient diagnoses, we therefore first develop algorithms, e.g., based on profile reviewing. These algorithms typically combine different types of information such as diagnosis codes and treatment data to increase specificity. In addition, we have conducted studies comparing incidences determined in GePaRD to those determined based on registries or other data sources, e.g. with respect to information on tumor stage [1]. Finally, as far as possible regarding the strict regulations for data privacy in Germany, we try to conduct validation studies that directly link data from GePaRD to other data sources. For example, this has successfully been completed for information on death and date of death where information in GePaRD was linked to the official mortality data [2].

[1] Oppelt KA, Luttmann S, Kraywinkel K, Haug U. Incidence of advanced colorectal cancer in Germany: comparing claims data and cancer registry data. BMC Med Res Methodol. 2019;19(1):142. DOI: 10.1186/s12874-019-0784-y.

[2] Langner I, Ohlmeier C, Zeeb H, Haug U, Riedel O. Individual mortality information in the German Pharmacoepidemiological Research Database (GePaRD): a validation study using a record linkage with a large cancer registry. BMJ Open. 2019;9(7):e028223. DOI: 10.1136/bmjopen-2018-028223

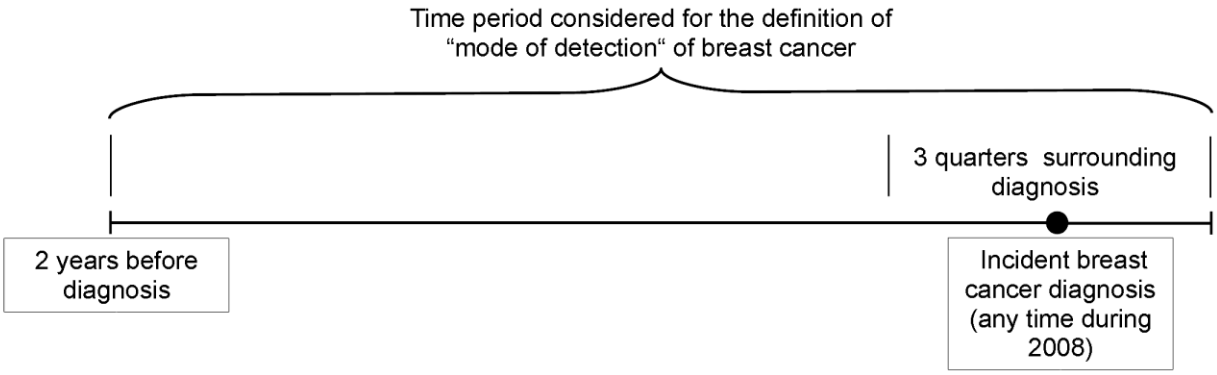

Figure A1 Visualization of the definition of mode of detection.

We classified a diagnosis as “screen-detected” if a screening mammography and a case conference were coded in the time period “two years before diagnosis until the quarter following the diagnosis”. At least one of these codes had to be recorded in the surrounding quarters of diagnosis (i.e., in the quarter before diagnosis, in the quarter of diagnosis or the following quarter). The quarter following the diagnosis was also considered because codes may be recorded with a certain delay (this has been observed in case profiles, even though very rarely).

The diagnosis was classified as “interval cancer” if a screening mammography was coded in the time period “two years before diagnosis until the quarter following the diagnosis”, and the criteria for “screen-detected” cancer were not fulfilled.

If no mammography screening was coded in the time period “two years before diagnosis until the quarter following the diagnosis” and the woman was eligible for screening (i.e. 50–69 years at diagnosis), this woman was classified as “unscreened but eligible”. The remaining patients were classified as “unscreened and ineligible”.

Table A1 Characterization of included breast cancer patients and description of initial treatment phase by stage at diagnosis

			Stage at diagnosis					
	All		No affected lymph nodes/distant metastases		Affected lymph nodes only		Distant metastases	
	10802	(100%)	8816	(81.6%)	1454	(13.5%)	532	(4.9%)
**Age at diagnosis**								
Mean age at diagnosis (SD)	62.8	(12.2)	62.8	(12.1)	61.2	(12.4)	65.2	(12.9)
<50 years at diagnosis	1750	(16.2%)	1419	(16.1%)	266	(18.3%)	65	(12.2%)
50-69 years at diagnosis	5950	(55.1%)	4910	(55.7%)	781	(53.7%)	259	(48.7%)
70-79 years at diagnosis	2119	(19.6%)	1702	(19.3%)	284	(19.5%)	133	(25.0%)
80+ years at diagnosis	983	(9.1%)	785	(8.9%)	123	(8.5%)	75	(14.1%)
**Mode of detection[a]**								
Screen-detected	2049	(19.0%)	1807	(20.5%)	206	(14.2%)	36	(6.8%)
Interval-detected	476	(4.4%)	392	(4.4%)	72	(5.0%)	12	(2.3%)
Unscreened (eligible)	3540	(32.8%)	2816	(31.9%)	510	(35.1%)	214	(40.2%)
Unscreened (not eligible)	4737	(43.9%)	3801	(43.1%)	666	(45.8%)	270	(50.8%)
**Breast surgery[b]**								
Breast conserving surgery	7518	(69.6%)	6438	(73.0%)	862	(59.3%)	218	(41.0%)
Radical breast surgery	3284	(30.4%)	2378	(27.0%)	592	(40.7%)	314	(59.0%)
Both types of surgery	1078	(10.0%)	822	(9.3%)	201	(13.8%)	55	(10.3%)
Two or more surgeries	2716	(25.1%)	2239	(25.4%)	387	(26.6%)	90	(16.9%)
**Neoadjuvant systemic therapy**								
Yes	954	(8.8%)	728	(8.3%)	109	(7.5%)	117	(22.0%)
**Adjuvant systemic therapy[c]**								
Cytostatic drugs	4446	(41.2%)	3186	(36.1%)	1023	(70.4%)	237	(44.5%)
Monoclonal antibody	96	(0.9%)	57	(0.6%)	17	(1.2%)	22	(4.1%)
Hormone therapy	5173	(47.9%)	4542	(51.5%)	362	(24.9%)	269	(50.6%)
**Radiotherapy[d]**								
Within ten months after breast surgery	8073	(74.7%)	6610	(75.0%)	1166	(80.2%)	297	(55.8%)
Before breast surgery	58	(0.5%)	21	(0.2%)	6	(0.4%)	31	(5.8%)

Abbreviations: SD = standard deviation

a Breast cancer was classified as "screen-detected" if a screening mammography and multidisciplinary case conference were coded in relevant time periods before and surrounding the diagnosis. It was classified as "interval-detected" if the woman had a screening mammography in the regular interval (two years) before diagnosis, but the criteria for "screen-detected" were not fulfilled. Patients without a screening mammography in the regular interval and aged 50–69 years at diagnosis were classified as unscreened, but eligible. The remaining patients were classified as "unscreened and ineligible". Some patients may be diagnosed, e.g., at age 70 and screened at age 69.

b Within one year after diagnosis. Mastectomy includes those with both types of surgery. "Two or more surgeries" refers to additional breast conserving surgery/mastectomy in the first year after the first surgery.

c This refers to adjuvant systemic therapy initiated within four months after breast surgery.

d This refers to radiotherapy initiated within ten months after breast surgery.

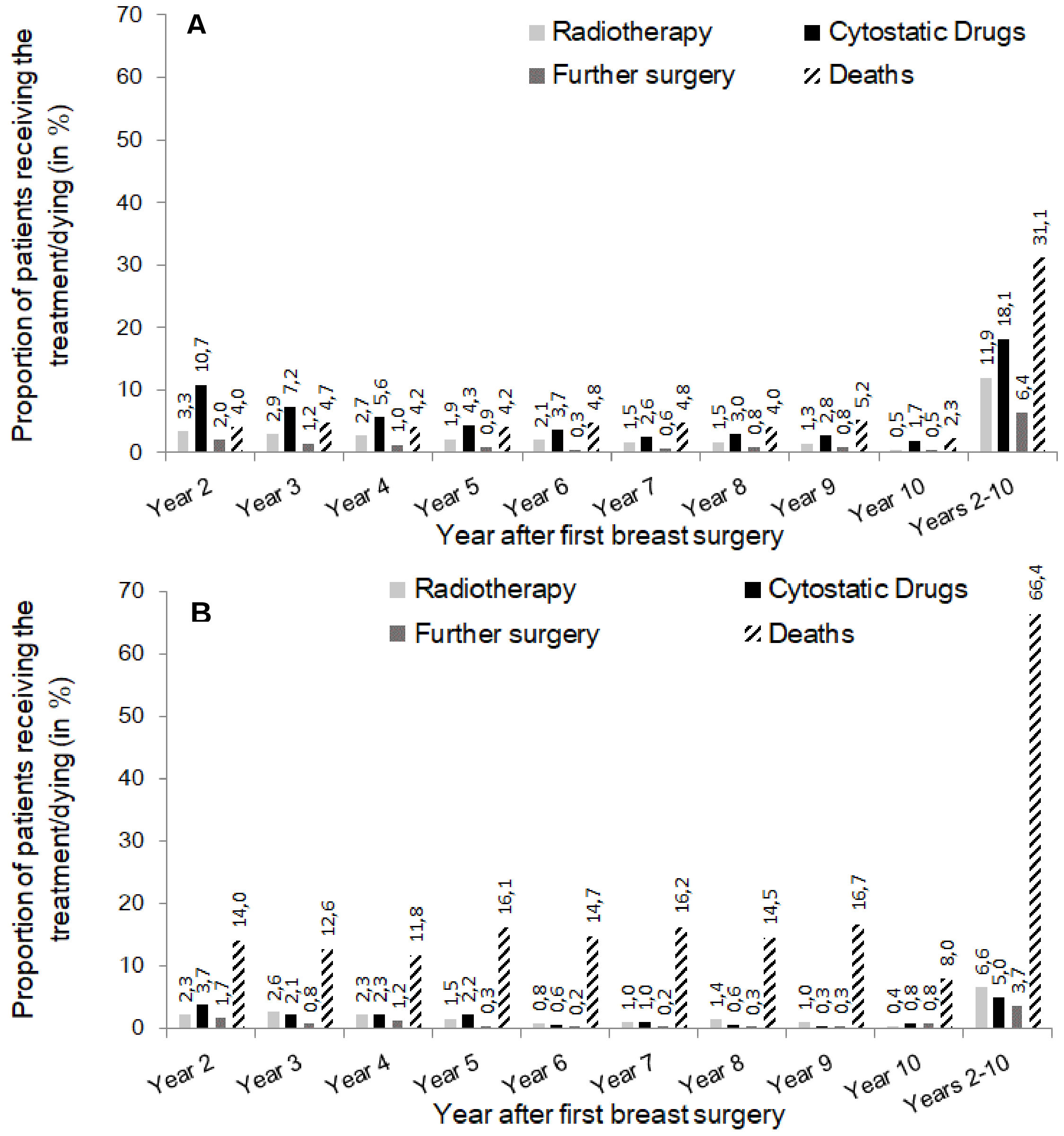

Figure A2 Long-term treatment patterns (radiotherapy, cytostatic drugs, further surgery) and deaths in the years 2–10 among included breast cancer patients stratified by age group (70-79 years (A) and 80 years and older (B) at diagnosis). Year ten is not a full year because observation ended on December 31, 2017.

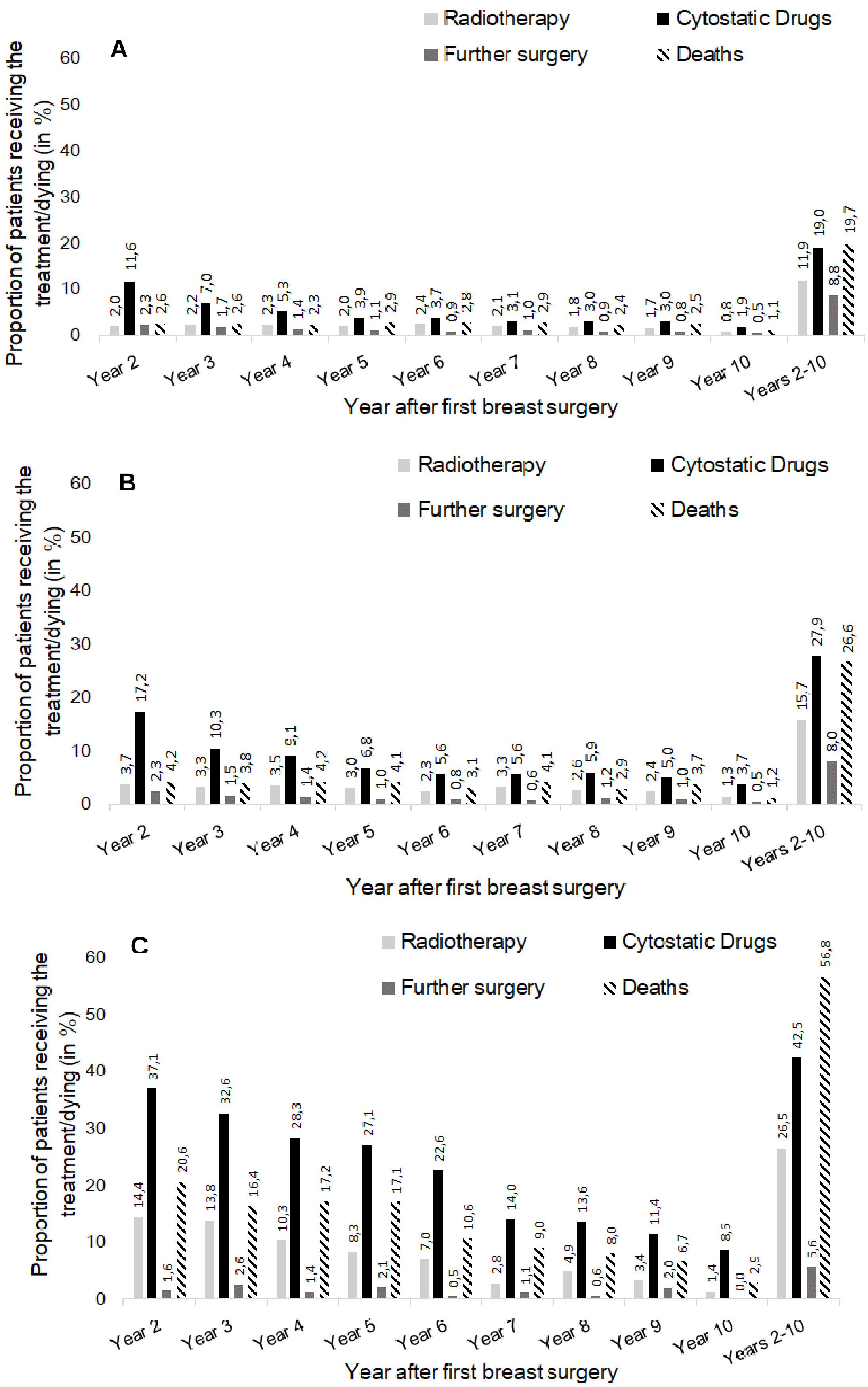

Figure A3 Long-term treatment patterns (radiotherapy, cytostatic drugs, further surgery) and deaths in the years 2–10 among all included breast cancer patients. Year ten is not a full year because observation ended on December 31, 2017.
